# Intraperitoneally Delivered Mesenchymal Stem Cells Alleviate Experimental Colitis Through THBS1-Mediated Induction of IL-10-Competent Regulatory B Cells

**DOI:** 10.3389/fimmu.2022.853894

**Published:** 2022-03-18

**Authors:** Jialing Liu, Xingqiang Lai, Yingying Bao, Wenfeng Xie, Zhishan Li, Jieying Chen, Gang Li, Tao Wang, Weijun Huang, Yuanchen Ma, Jiahao Shi, Erming Zhao, Andy Peng Xiang, Qiuli Liu, Xiaoyong Chen

**Affiliations:** ^1^ The Biotherapy Center, The Third Affiliated Hospital, Sun Yat-sen University, Guangzhou, China; ^2^ Center for Stem Cell Biology and Tissue Engineering, Key Laboratory for Stem Cells and Tissue Engineering, Ministry of Education, Sun Yat-sen University, Guangzhou, China; ^3^ Department of Cardiology, The Eighth Affiliated Hospital, Sun Yat-sen University, Shenzhen, China; ^4^ Department of Biochemistry, Zhongshan School of Medicine, Sun Yat-sen University, Guangzhou, China; ^5^ Department of Pathophysiology, Zhongshan School of Medicine, Sun Yat-sen University, Guangzhou, China

**Keywords:** mesenchymal stem cells, experimental colitis, regulatory B cells, THBS1, TGFβ (transforming growth factor β)

## Abstract

Mesenchymal stem cells (MSCs) show promising therapeutic potential in treating inflammatory bowel disease (IBD), and intraperitoneal delivery of MSCs have become a more effective route for IBD treatment. However, the underlying mechanisms are still poorly understood. Here, we found that intraperitoneally delivered MSCs significantly alleviated experimental colitis. Depletion of peritoneal B cells, but not macrophages, clearly impaired the therapeutic effects of MSCs. Intraperitoneally delivered MSCs improved IBD likely by boosting the IL-10-producing B cells in the peritoneal cavity, and a single intraperitoneal injection of MSCs could significantly prevent disease severity in a recurrent mouse colitis model, with lower proinflammation cytokines and high level of IL-10. The gene expression profile revealed that thrombospondin-1 (THBS1) was dramatically upregulated in MSCs after coculture with peritoneal lavage fluid from colitis mice. Knockout of THBS1 expression in MSCs abolished their therapeutic effects in colitis and the induction of IL-10-producing B cells. Mechanistically, THBS1 modulates the activation of transforming growth factor-β (TGF-β), which combines with TGF-β receptors on B cells and contributes to IL-10 production. Blocking the interaction between THBS1 and latent TGF-β or inhibiting TGF-β receptors (TGF-βR) significantly reversed the THBS1-mediated induction of IL-10-producing B cells and the therapeutic effects on colitis. Collectively, our study revealed that intraperitoneally delivered MSCs secreted THBS1 to boost IL-10^+^Bregs and control the progression and recurrence of colitis, providing new insight for the prevention and treatment of IBD.

**Graphical Abstract:**
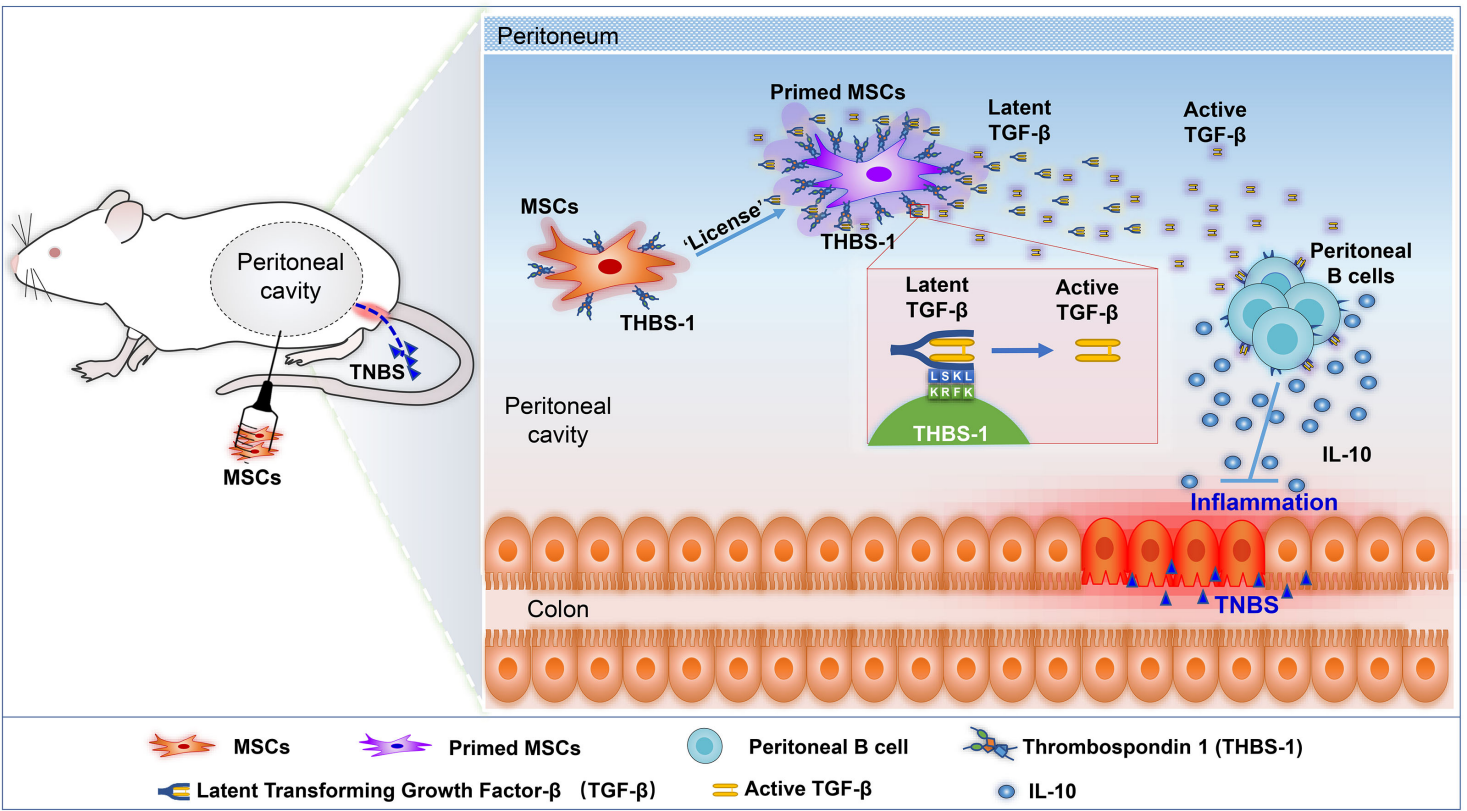


## Introduction

Inflammatory bowel disease (IBD), mainly including ulcerative colitis (UC) and Crohn’s disease (CD), has chronic recurrent inflammation in the gastrointestinal tract, manifesting as abdominal pain, diarrhoea, rectal bleeding, and even fatigue and weight loss. IBD is an immune-mediated inflammatory disease, especially persistent local inflammation in the intestine (1, 2). To date, no specific therapies are currently available for IBD patients, and the strategies for IBD treatment are still immunosuppression, with the main purpose of controlling inflammation, thereby providing short-term or long-term relief and reducing the risk of complications (3). Thus, the drugs used to treat IBD mainly include glucocorticoids, immunosuppressants, and biological agents. However, these treatments are limited by serious side effects or loss of sustained response to treatment over time (4). Systemic immunosuppressive therapy usually causes serious consequences, such as elevated blood glucose levels and an increased risk of osteoporosis and infection (5). Biological drugs mainly targeting the TNF-α signalling pathway or its components have been used clinically and proven to alleviate IBD, such as anti-TNF-α antibodies, but almost one-third of IBD patients showed no improvement and even loss of sustained response over time (6–8). Therefore, new and effective therapy for IBD is still urgently needed.

Mesenchymal stem cells (MSCs), a kind of adult stem cell, were first found in bone marrow. Subsequently, MSCs are reported to reside in almost all tissues and organs, including adipose tissue, dental tissues, dermis, spleen, lung, circulating blood, and foetal tissue such as umbilical cord, placenta, and foetal membrane (9). MSCs have become an excellent mediator in tissue repair and regeneration with many advantages: MSCs can be recruited to sites in damaged or inflamed tissues and exhibit the potential to differentiate into other cell types and modulate the microenvironment for tissue repair. Moreover, MSCs have become a promising treatment for autoimmune diseases, including IBD, due to their immunomodulatory effects, such as inhibiting the T cell response, inducing regulatory T cells and B cells, and modulating NK cells, macrophages, dendritic cells, and other innate immune cells (10–14). A large number of clinical trials have confirmed the safety and efficiency of MSC-based therapy for IBD patients, even those who do not respond to conventional or biological treatments (15, 16). Systemic infusion of MSCs is still a common means for IBD treatment; however, an increasing number of studies have reported that intravenous delivery of MSCs yields unsatisfactory therapeutic effects (17–19). For example, in a phase I–II clinical study that enrolled 13 patients with severe CD, only one patient achieved clinical remission (20). Furthermore, the local intralesional injection of MSCs has been gradually used and has shown promising outcomes in repairing anal fistulas (21), one complication of CD. In a phase 3 randomized and double-blind controlled trial, local injection of MSCs was demonstrated to be an effective and safe treatment for complex perianal fistulas in patients with CD (22). However, local injection might not be a convenient method for 70% of IBD patients who do not have fistulas (23). New methods for delivering MSCs in the clinic still need to be explored.

Preclinical studies have demonstrated that intraperitoneal injection of MSCs is a better way to treat colitis, with higher efficiency than intravenous infusion (24), with a higher survival rate, less weight loss and quick weight gain, and better mucosa recovery. However, the underlying mechanisms are still not well known. A previous study reported that intraperitoneally delivered MSCs rarely migrated into the intestinal tract, and they were likely to stay in the peritoneal cavity to modulate immune cells (25). Peritoneal cells were reported to mediate immune responses, such as cross-protection against influenza A virus (26). Moreover, adoptive transfer of peritoneal cells could significantly improve colitis (27), suggesting that modulating peritoneal cells might be an effective method for IBD treatment. Therefore, we hypothesized that intraperitoneal administration of MSCs may modulate immune cells in the intraperitoneal cavity, thereby playing an important role in alleviating IBD. Here, we explored the underlying mechanisms and targeted the cell population by which intraperitoneal delivered MSCs alleviated IBD. We found that intraperitoneally delivered MSCs significantly alleviated experimental colitis. Intraperitoneal delivery of MSCs improved IBD, likely by boosting IL-10-producing B cells in the peritoneal cavity. Intraperitoneal injection of MSCs also alleviated the recurrence of colitis. Importantly, we determined that MSC-derived thrombospondin-1 (THBS1) cells modulate the activation of TGF-β, which combines with TGF-β receptors on B cells and contributes to IL-10 production. Our study further confirmed that intraperitoneal administration might be the optimum MSC delivery route for the treatment of colitis, not only reducing the severity of disease but also preventing the recurrence of disease. Moreover, we reveal the critical role of THBS1, TGF-β, and regulatory B cells in alleviating colitis, providing new insight for the prevention and treatment of IBD.

## Materials and Methods

### Isolation and Characterization of hMSCs

MSCs were isolated from bone marrow samples obtained from healthy donors. The study followed the Declaration of Helsinki protocols with informed consent, and the protocol was performed according to our previous study (28). Briefly, mononuclear cells were obtained by Ficoll-Hypaque (Amersham Biosciences, Uppsala, Sweden) density gradient centrifugation and seeded into 75-cm^2^ flasks (CellBIND, Corning, New York, Corning, USA) at 1 × 10^5^/cm^2^ density. When the cells had grown to 80% confluence, they were isolated with trypsin-EDTA and designated as the first generation. The cells were passaged at a ratio of 1:3. MSCs were characterized as follows: (1) plastic adhesion growth; (2) expression of surface markers: more than 95% of the cells expressed CD105, CD73, and CD90; less than 2% of the cells expressed CD45, CD34, CD11b, CD19, and HLA-DR; and (3) ability to differentiate into osteoblasts, adipocytes, and chondroblasts *in vitro*. As shown in **Figure S1**, the isolated MSCs met the identification standards, and the well-characterized 5th–6th passage MSCs were used for both *in vivo* and *in vitro* experiments.

### TNBS-Induced Colitis

Colonic inflammation was induced in male Balb/c mice by intrarectal administration of the hapten substances trinitrobenzenesulfonic acid (TNBS, Sigma-Aldrich, St. Louis, MO, USA) and ethanol as described (29). Briefly, a 1.5 × 1.5 cm area of the skin was scraped between the back shoulders, avoiding licking by the mice. A total of 150 μl of 1% (WT/VOL) TNBS presensitized solution was applied to the shaved area, performing a haptenized immune response for 7 days (30). The body weight was recorded, and the mice that had abnormal body weight loss (loss more than 5% of the initial weight) were removed from the experiments. Subsequently, food (but not water) was withdrawn for 24 h, and then mice were anaesthetized and placed upside down. A 0.7-mm disposable epidural catheter was used to clyster from the anus into the colon 4 cm, and 100 μl 2.5% (WT/VOL) TNBS (mixed with 5% TNBS and anhydrous ethanol in a volume ratio of 1:1) was injected. The mice were maintained in a vertical position for 30 s to ensure the proper distribution of TNBS within the colon. This time point was set as Day 0. The studies were approved by the Animal Care and Use Committee of Sun Yat-sen University (SYSU-IACUC-2020-0761).

For MSC transplantation, the 5th–6th passage cultured MSCs were digested into single cells and resuspended in PBS for counting. Then, the concentration of MSCs was adjusted to 1 × 10^7^/ml, and 1 × 10^6^ MSCs suspended in 100 μl PBS was transplanted intraperitoneally. PBS alone was used as a vehicle control. All injections were administered 24 h before TNBS administration. For TGF-βR blockade, LY364947 (1 mg/kg; Selleck Chemical, Houston, USA) was intraperitoneally administered 3 times a week to block TGF-βR before MSC transplantation.

### B Cell and MSC Culture Assay

Mouse peritoneal cavity cells were isolated following a previously published protocol (31). Briefly, scissors and forceps were used to cut the outer skin of the peritoneum of sacrificed mice. Four millilitres of RPMI 1640 medium was injected into the peritoneal cavity, followed by gentle massage of the abdomen, and cells were harvested by collecting peritoneal lavage fluid. Cell counting was performed by a cell counter, and then peritoneal cavity cells were cocultured with MSCs at a ratio of 5:1 or cultured alone as a control. For THBS1 blocking experiments, LSKL peptide (10 μM, MedChemExpress, Princeton, NJ, US) was added to the coculture systems. For the intracellular IL-10 cytokine assay, the Leukocyte Activation Cocktail (2 µl/ml, BD Biosciences, Franklin Lakes, NJ, USA), a ready-to-use polyclonal cell activation mixture, was added in the last 6 h before cell harvesting.

### THBS1 Knockout Plasmid Construction

Single-guide RNAs targeting the human THBS1 locus (exon 1) were designed using E-CRISP tools (sequences shown in **Table 1**). Annealed guide oligos were chosen and cloned into the CRISPR–Cas9 expression vector LentiCRISPR-2A-GFP by T4 DNA ligase (TaKaRa Bio, Kusatsu, Japan). The constructed knockout vector and lentiviral packaging vectors pMD2. G and psPAX2 (Addgene, Watertown, MA, USA) were transfected into HEK-293T cells with MegaTran 1.0 (OriGene, Rockville, MD, USA) at a 4.5-μg concentration. New antibiotic-deficient complete medium was replaced after transfection for 24 h. The viral supernatants were collected at 48 and 72 h, and MSCs were transfected with the viral supernatant:medium = 1:1 (VOL). The green fluorescence expression of MSCs was observed after 48 h of infection, and THBS1-KO MSCs were purified by flow cytometry.

**Table 1:** List of primer sequences.

Name	Primers
GAPDH F	5′-atcactgccacccagaag-3′
GAPDH R	5′-tccacgacggacacattg-3′
TNF-*α F*	5′-catggatctcaaagacaaccaa-3′
TNF-α R	5′-ctcctggtatgacattgcaaat-3′
IL-6 F	5′-ccaccgccttccctacttca-3′
IL-6 R	5′-tggtccttagccactcctgc-3′
IFN-γ F	5′-cgctacacactgcatcttgg-3′
IFN-γ R	5′-gctttcaatgactgtgccgt-3′
THBS1 sg F	5′-caccggcacgctgctggccctggag-3′
THBS1 sg R	5′-aaacctccagggccagcagcgtgcc-3′

### B Cell Depletion in Mice

Peritoneal cavity B cells were depleted following the protocol of a previous study (32). Briefly, mice were intraperitoneally injected with 250 μl (1 μg/μl) anti-mouse CD20 monoclonal (Clone SA271G2, BioLegend, San Diego, CA, USA) antibody or purified rat IgG isotype control 3 days before TNBS administration to deplete B cells. Peripheral blood cells and intraperitoneal cells were collected at Day 5 after administration. The samples were incubated with anti-mouse CD19 antibody. CD19^+^ B cells in the peritoneal cavity and peripheral blood were detected by flow cytometry.

### Macrophage Cell Depletion in Mice

Peritoneal cavity macrophages were depleted following a previous study (33). Briefly, mice were injected with clodronate liposomes, an effective macrophage scavenger, or PBS liposomes as a negative control. Each mouse was intraperitoneally injected with 150 μl (5 mg/ml) clodronate liposomes or liposomes (PBS) (Liposoma BV, Amsterdam, Netherlands) as recommended by the manufacturer. After 5 days of administration, abdominal cavity cells were collected and incubated with anti-mouse F4/80 antibody. Mouse F4/80^+^ macrophage cells were detected by flow cytometry.

### Colitis Assessment

Animal survival, mobility, and faecal consistency were recorded daily. Mouse body weight was monitored daily, and mice were euthanized on the third day after the induction of colitis (the peak of the disease). The colon was collected without the caecum, measured for length, and assessed for macroscopic or microscopic damage. Disease activity and scores were assessed as described. The severity of colitis was assessed according to the colitis score, the degree of macroscopic damage, the length of colon, and the degree of histological damage (29).

### Flow Cytometry

The cells were collected into flow tubes, and the cell precipitate was obtained by centrifugation at 1,500 rpm for 5 min. The cells were washed twice with PBS. Each sample was diluted and resuspended with flow buffer:flow antibody = 50:1 (VOL) and incubated at 4°C for 30 min in the dark. The cells were washed with PBS, centrifuged at 1,500 rpm for 5 min, and then fixed with 4% paraformaldehyde (Sigma-Aldrich, USA) for 20 min. The cells were washed with PBS and centrifuged. After this was repeated twice, each tube was resuspended in 100 μl total volume (1% saponin (Sigma-Aldrich, US): PBS: flow cytometry antibody = 20:80:2 (VOL)) in a vortex oscillator and incubated for 30 min with protection from light at 4°C. After washing twice with PBS, flow cytometry was used for analysis and detection. The antibodies were used as described in **Table S1** and purchased from BD Biosciences. The data were detected by a CytoFLEX Flow Cytometer (Beckman, Indianapolis, IN, USA) and analysed by FlowJo 10.6.2 Software (FlowJo LLC, Ashland, OR, USA).

### Histological Examination

The animals were euthanized on Day 2 to study the inflammatory response to colitis. The colon was washed with PBS, fixed with 4% paraformaldehyde, and paraffin-embedded (Sigma-Aldrich, USA). The tissue sections were stained with haematoxylin and eosin (HE) and evaluated by two investigators in a blinded manner. Histological changes in TNBS-induced colitis mice were quantified (range 0 to 4) using the Macpherson and Pfeiffer histopathological grading system (34). In brief, the same histological finding score as normal mice was 0, with mild mucosal and/or submucosal inflammatory infiltration and oedema, punctured mucosal erosion, and intact mucosal muscularis.

### ELISA

The peritoneal fluid of the mice in the normal mouse control group and model groups was collected by sterile syringe extraction into tubes and centrifuged at 2,500 rpm/min at 4°C for 20 min. The supernatant was aspirated for analysis. Enzyme-linked immunosorbent assay (ELISA) was performed according to the manufacturer’s protocol (mouse IL-6, TNF-α ELISA kit from Neobioscience, Shenzhen, China; mouse IL-10, IFN-γ ELISA Kit from R&D Systems, Minneapolis, MN, USA). Briefly, the collected supernatants were added to a ready-to-use 96-well plate, which had been coated with IL-6, TNF-α, IL-10, or IFN-γ antibodies and detected *via* the ELISA sandwich technique (35). Finally, the absorbance of each well was determined by a microplate reader (Thermo Scientific, Vantaa, Finland).

### RT-qPCR

Mouse colon tissues were collected at the same position and then weighed, and the tissue samples were frozen in liquid nitrogen. The tissues were ground with liquid nitrogen (36). According to the manufacturer’s recommendation, total RNA was extracted from tissues using TRIzol reagent (Invitrogen, Carlsbad, CA, USA) and reverse transcribed into cDNA using NovoScript Plus All-in-one 1st Strand cDNA Synthesis SuperMix (gDNA Purge) (Novoprotein, Suzhou, China). RT-qPCR was performed using LightCycler 480 SYBR Green I Master Mix (Roche Diagnostics GmbH, Mannheim, Germany). Each 20-μl RT-qPCR contained 1 μl cDNA, 1 μM forward and reverse primers, and 10 μl 2× SYBR Green Master Mix. The mRNA expression levels of target genes included TNF-α, IFN-γ, IL-6, and GAPDH. CT values were calculated in relation to GAPDH CT values by the 2^-△△CT^ method (37), in which △Ct was the difference between the Ct value of genes and the Ct value of GAPDH. All assays were repeated in three independent experiments. The primers are described in **Table 1**.

### Differential Expression Analysis

R language (4.0) was used for gene difference analysis. To avoid false-positives, pseudogenes and genes with TPM values less than 5 in all cell lines were removed. The original data were then subjected to background correction, normalization, and expression calculation by using the R package “DESeq2”. Based on the significance threshold of p value <0.05 and log2(Fold Change) > 1 (upregulated) or log2(Fold Change) < −1 (downregulated), the differentially expressed genes (DEGs) were identified. Volcano plots and heatmaps were drawn by using the ggplot2 package in R.

### GO Pathway Enrichment Analysis of DEGs

Gene Ontology (GO) enrichment analysis was conducted by using the cluster Profiler R package with the upregulated genes, downregulated genes, and all the DEGs from biological processes, molecular functions, and cell components. Fisher’s exact test was used to calculate the significance, and a bubble diagram was used for display.

### Statistics

All data are expressed as the mean ± SD. GraphPad Prism 9 Software (CA) was used for statistical analysis, and a statistical comparison was made using the two-tailed Student’s t test between two groups or one-way ANOVA for multigroup comparison. The difference was considered statistically significant at *p* ≤ 0.05.

## Results

### MSC Intraperitoneal Treatment Alleviated the TNBS-Induced Colitis Model

**Figure 1:**
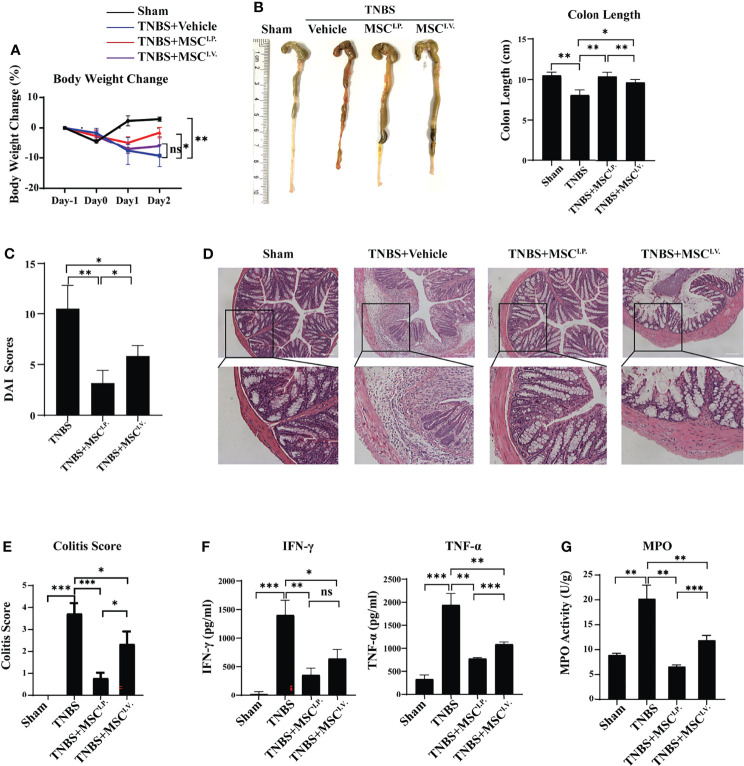
Intraperitoneal injection of MSCs mitigates TNBS-induced colitis in mice. MSCs were administered intravenously or intraperitoneally to IBD mice, and their therapeutic effects were evaluated. **(A)** Comparison of body weight loss after TNBS induction. **(B)** Comparison of colon lengths after TNBS induction. **(C)** The disease activity indices (DAI) were evaluated based on scores of percentage weight loss, stool consistency, and bleeding. **(D)** Representative H&E staining of colon cross sections and **(E)** colitis histological scoring at Day 2 after TNBS administration. **(F)** Secretion of cytokines in colon homogenates and **(G)** MPO activity. Statistical significance was determined by unpaired two-sided test as the means ± SD, n ≥ 3. not significant (ns) p≥0.05, *p < 0.05, **p < 0.01, ***p < 0.001, ****p < 0.0001. IL-, interleukin; TNF, tumour necrosis factor.

2,4,6-Trinitrobenzenesulfonic acid (TNBS)-induced experimental colitis is a well-documented murine model for human IBD, with similar morphological and histopathological features. Therefore, we used TNBS colitis to investigate the underlying mechanisms and targeted cell population in MSC-alleviated IBD. The TNBS-colitis mouse model was established as previously described (3). To investigate the effect of MSCs on IBD, MSCs were intravenously or intraperitoneally injected to treat TNBS-induced colitis. Weight change, colon length, disease activity index (DAI), and histological examination were measured and observed at Day 2 after MSC administration. The results showed that the MSC intravenous injection group (MSC^I.V.^) had improved colon length compared with the TNBS treatment group (TNBS), and mice from the MSC intraperitoneal injection group (MSC^I.P.^) had significantly lower decreases in both body weight and colon length than MSC^I.V.^ (**Figures 1A, B**). Additionally, colitis mice from MSC^I.P.^ had lower DAI scores (**Figure 1C**). Histopathological staining of colon slices showed that the normal group (Sham) had a normal intestinal cell structure and epithelial layer with the presence of goblet cells in straight ducts, and the TNBS-treated group showed severe inflammation with transmural necrosis, oedema, and inflammatory cell infiltration. Slices from MSC^I.P.^ had less inflammatory cell infiltration within the lamina propria than MSC^I.V^ (**Figures 1D, E**). Meanwhile, we investigated the inflammatory factors IFN-γ and TNF-α in colon tissues, and ELISA results indicated that MSC^I.P.^ had fewer inflammatory cytokines than the MSC^I.V.^ group (**Figure 1F**). In addition, we investigated MPO activity in the injured colon, which reflected inflammatory cell infiltration. Our results showed that MPO activity was significantly downregulated in the MSC^I.P.^ group (**Figure 1G**). Taken together, the results confirmed that intraperitoneally delivered MSCs provided more effective protection in colitis mice than intravenously delivered MSCs.

### Peritoneal B Cells Involved Critically in MSC-Mediated Alleviation for Colitis

**Figure 2:**
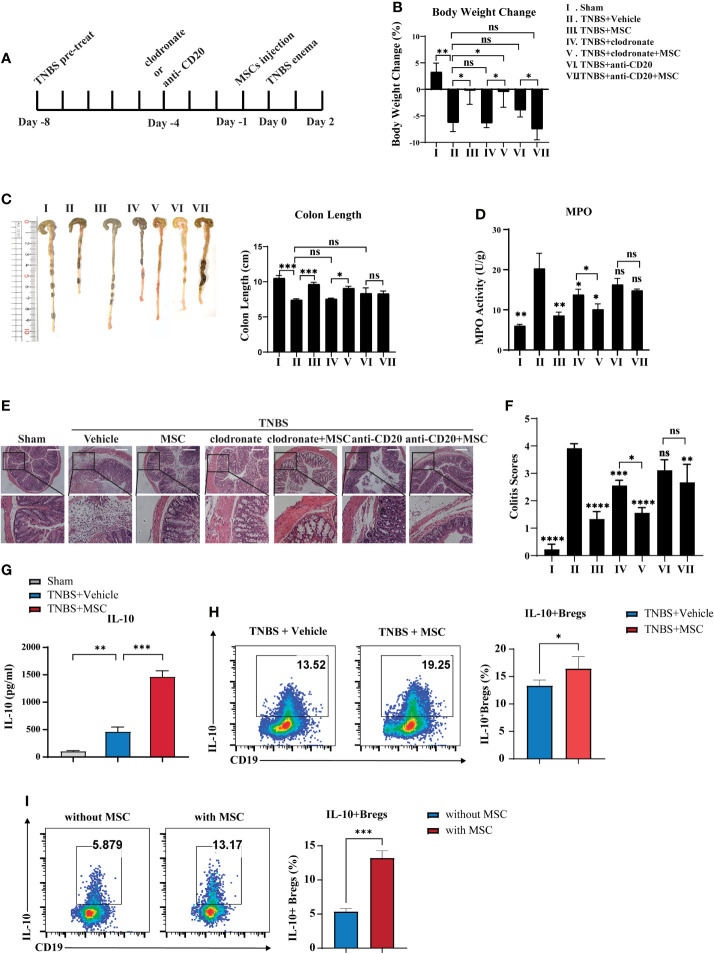
B cells are critically involved in MSC-mediated effects on attenuating colitis. Clodronate liposomes were used to deplete intraperitoneal macrophages. Anti-mouse CD20 monoclonal antibodies were used to intraperitonealize B cells before TNBS enema for 96 h with or without MSC administration, following the workflow **(A)**. Then, their effects on MSCs treating colitis were investigated. Comparison of body weight loss **(B)**, colon length **(C)**, MPO activity **(D)**, histological examination of colon cross sections with H&E staining **(E)**, and histological scoring **(F)**. Additionally, the alteration of IL-10 secretion levels in peritoneal fluid was determined by ELISA **(G)**, and the alteration of peritoneal Bregs was detected by flow cytometry **(H)**. Moreover, peritoneal cells were cocultured with MSCs *in vitro*, and IL-10^+^Bregs after coculture with or without MSCs were detected by flow cytometry **(I)**. Statistical significance was determined by unpaired two-sided Student’s t test. Data are presented as the means ± SD, n ≥ 3. not significant (ns) p≥0.05, *p < 0.05, **p < 0.01, ***p < 0.001, ****p < 0.0001. All groups were arranged as follows: Sham, the normal group; TNBS+Vehicle, TNBS-induced group with PBS treatment; TNBS+MSCs, TNBS induced with MSC intraperitoneal injection group; TNBS+clodronate, clodronate liposome treatment to macrophage depletion group; TNBS+clodronate+MSCs, clodronate liposome treatment and MSC injection group; anti-CD20, anti-mouse CD20 monoclonal antibody (mAb) treatment group; anti-CD20+MSC, anti-mouse CD20 mAb treatment, and MSC injection group.

Previous studies have reported the important role of peritoneal cells in controlling colitis; therefore, we speculated that MSCs might modulate peritoneal cells to exert their therapeutic effects. Considering that B cells and macrophages are two major types of cells in the peritoneal cavity (31), we investigated whether B cells or macrophages participate in MSC-mediated colitis alleviation. Anti-CD20 monoclonal antibody and clodronate liposomes were applied separately for B-cell or macrophage depletion in the peritoneal cavity before MSC injection (**Figure 2A**). The results showed that B cells or macrophages were dramatically reduced in the peritoneal cavity (**Figure S2**). Compared to the control group, clodronate liposome administration reduced the MPO activity and DAI of colitis but not the weight loss or colon length, while injection of the anti-CD20 monoclonal antibody had little effect on colitis (**Figures 2B–F**). These results suggested that peritoneal macrophages might be involved in the development of colitis, but MSCs could still improve colitis in the absence of macrophages in the peritoneal cavity. Additionally, when B cells were depleted in the peritoneal cavity, MSC-mediated colitis alleviation was dramatically abolished, indicating that MSCs are most likely to protect against colitis *via* peritoneal B cells. Combined with our result that the concentration of IL-10 in peritoneal lavage fluid was significantly elevated in the MSC treatment group (**Figure 2G**), we speculated that MSCs might induce IL-10-competent regulatory B cells (Bregs). Thus, we detected IL-10^+^ Bregs in the peritoneal cavity and found that IL-10^+^B cells were increased when MSCs were administered (**Figure 2H**). To further confirm this, peritoneal cells were collected and cocultured with MSCs *in vitro*; similarly, MSCs could effectively induce IL-10^+^Bregs (**Figure 2I**). In addition, we examined the cell surface phenotypes of these MSC-induced Bregs and found that these IL-10^+^Bregs expressed high levels of CD5, CD24, CD38, CD43, IgM, and CD1d but low expression of IgD (**Figure S3**), suggesting that these IL-10^+^ Bregs might be B1 cells, a unique B cell subpopulation residing mainly in the peritoneal cavity. Taken together, our results revealed that intraperitoneally delivered MSCs alleviated colitis in mice, likely by boosting IL-10-producing B cells in the peritoneal cavity.

### Intraperitoneal Injection of MSCs Alleviated the Recurrence of Mouse Colitis

**Figure 3:**
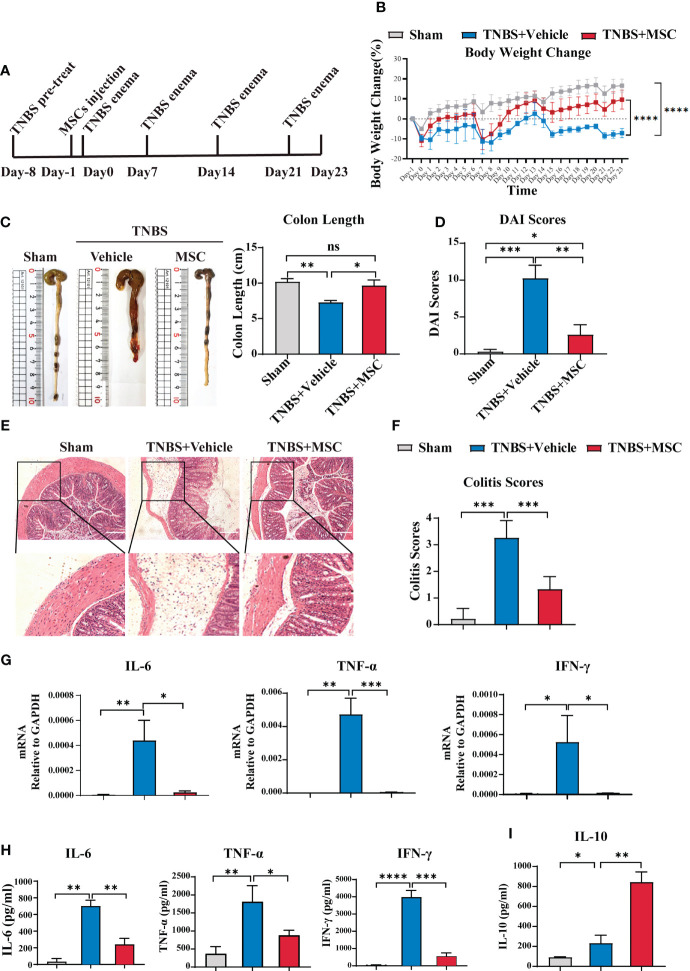
Intraperitoneal delivery of MSCs effectively prevented the recurrence of colitis. A mouse recurrence IBD model was established by four administrations of TNBS (100 μl enema per mouse every 7 days for up to 4 weeks). A single intraperitoneal injection of MSCs was delivered, and the protection of MSCs was investigated. Mice were euthanized at 23 days and collected for analysis. **(A)** Mouse IBD recurrence model schematic illustration. **(B)** Comparison of body weight loss. **(C)** Comparison of colon length. **(D)** Comparison of DAI combined scores of percentage weight loss, stool consistency, and bleeding. **(E)** Histological examination of colon cross sections *via* H&E staining and **(F)** histological scoring. **(G)** Colon tissue was collected, and total RNA was extracted. The expression level of proinflammatory cytokines was evaluated in the colons of mice from the sham, TNBS-induced, and MSC treatment groups through RT–PCR. **(H)** Colon tissue was collected, and the expression levels of proinflammatory cytokines in colon homogenate were detected by ELISA. **(I)** Peritoneal fluid was harvested, and the IL-10 level was determined by ELISA. Data are presented as the means ± SD, n ≥ 3. not significant (ns) p≥0.05, *p < 0.05, **p < 0.01, ***p < 0.001, ****p < 0.0001.

B cells in the abdominal cavity mediate immune tolerance *via* IL-10 production and prevent miscarriage during pregnancy (38). Considering that IBD is a relapsing inflammatory disorder, we further evaluated whether intraperitoneally delivered MSC-induced IL-10 secretion of peritoneal B cells could prevent colitis recurrence *via* a relapsing mouse model of colitis (**Figure 3A**), established by four administrations of TNBS as previously described (39). As shown in **Figure 3B**, the weight of mice in the control group increased daily. Every TNBS injection caused weight loss in mice. With the increase in the number of TNBS injections, the weight difference between colitis mice and control mice increased. The weight of the colitis mice was always lower than the baseline level at the beginning. Interestingly, a single injection of MSCs mediated a long-lasting effect in attenuating colitis, exhibiting less weight loss during the recurrence of colitis (**Figure 3B**). Although each TNBS injection still caused weight loss in mice from the MSC injection group, the weight loss decreased over time. Moreover, the weight of these mice increased gradually, showing a trend similar to that of the control group (**Figure 3B**). The colon length (**Figure 3C**), DAI (**Figure 3D**), and histopathological examination (**Figures 3E, F**) further confirmed that a single intraperitoneal injection of MSCs could significantly alleviate the recurrence of colitis. Histopathological results revealed that MSCs mitigated intestinal injury and inflammation; consistently, proinflammatory cytokines, playing essential roles in the induction and maintenance of inflammation in the intestine, such as TNF-α, IFN-γ, and IL-6, were significantly inhibited in colon tissue by intraperitoneal treatment with MSCs (**Figure 3G**). In addition, the alteration of cytokines was also detected by ELISA. MSC administration not only reduced the levels of TNF-α, IFN-γ, and IL-6 inflammatory colon tissues but also maintained a higher IL-10 level in intraperitoneal fluid (**Figures 3H, I**). Briefly, our results demonstrated that intraperitoneally delivered MSCs mediated a long-lasting effect against the recurrence of colitis.

### Coculture With Peritoneal Lavage Fluid From Colitis Mice Significantly Enhanced the Expression of THBS1 in MSCs

**Figure 4:**
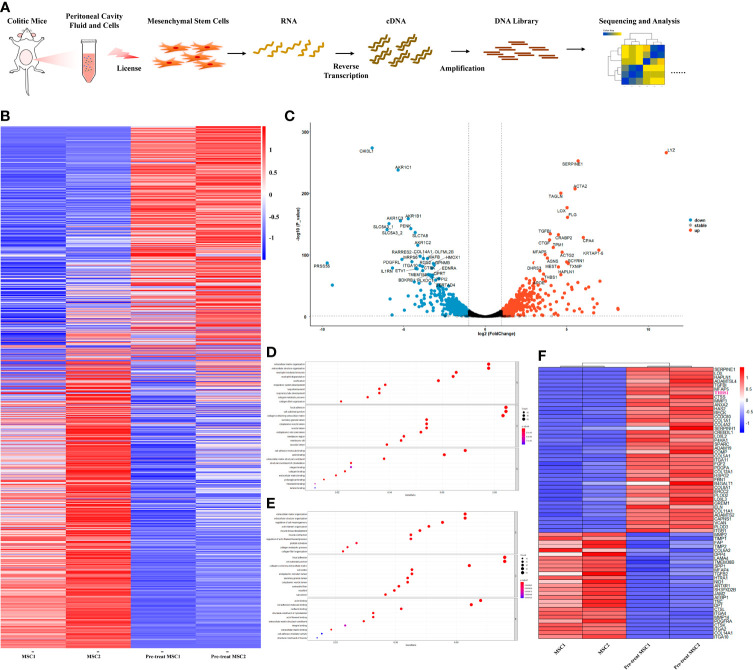
Peritoneal lavage fluid-primed MSCs enhanced the expression of expression. Peritoneal lavage fluid (including peritoneal cells) was collected from colitis mice and then cocultured with MSCs; simultaneously, MSCs cultured alone were used as a control. Next, the gene expression profile of MSCs was obtained by bulk RNA sequencing for further analysis. **(A)** Overview of the workflow. **(B)** Heatmap visualization for total included genes in pretreated MSCs compared to control MSCs. **(C)** Volcano plots for the distribution of differentially expressed genes (DEGs) from the comparison between primed MSCs and control MSCs. **(D)** GO enrichment analysis of all DEGs from the comparison between primed MSCs and control MSCs. **(E)** GO enrichment analysis of the upregulated DEGs from the comparison between primed MSCs and control MSCs. **(F)** Heatmap visualization for DEGs clustered in the extracellular matrix organization pathway.

Previous studies have shown that the therapeutic effect of MSCs mainly depends on their immunomodulation, which is ‘licensed’ by the microenvironment (40). To further explore how MSCs modulate IL-10 secretion by peritoneal B cells, we investigated the alteration in the gene expression profile of MSCs. Peritoneal lavage fluid (including peritoneal cells) was collected from colitis mice and then cocultured with MSCs; simultaneously, MSCs cultured alone were used as a control. Next, the gene expression profile of MSCs was obtained by bulk RNA sequencing, as shown in the workflow (**Figure 4A**). Compared to control MSCs, IBD mouse peritoneal lavage fluid stimulation significantly altered the gene expression of MSCs (**Figure 4B**), with 535 upregulated genes and 538 downregulated genes. Furthermore, a volcano plot was generated for a quick visual identification of genes with large fold changes that were also statistically significant to identify some biologically significant genes, and PRSS35, IL1RN, DLFML2B, HMOX1, GPNMB, MTHFD2, THBS1, etc., were significantly changed (**Figure 4C**). Gene Ontology (GO) analysis was carried out with all the DEGs and the upregulated genes to gain insight into the biological significance of the alterations. The results showed that the alterations in primed MSCs mainly manifested as changes in cell structure, extracellular matrix, and cell metabolism (**Figures 4D, E**). To further explore how MSCs regulate B cells, we focused on extracellular matrix alterations, which have been reported to modulate tissue repair and immunomodulation (41). The heatmap showed the main genes that were dramatically altered in the extracellular matrix (**Figure 4F**), and we found that THBS1, an adhesive glycoprotein that mediates cell-to-cell and cell-to-matrix interactions, was upregulated in MSCs licensed by peritoneal lavage fluid from colitis mice. Considering that THBS1 could trigger IL-10 production in macrophages (42), we speculated that THBS1 might participate in MSC-mediated effects on colitis.

### MSCs Induced IL-10-Producing B Cells and Alleviated Colitis in Mice Through THBS1

**Figure 5:**
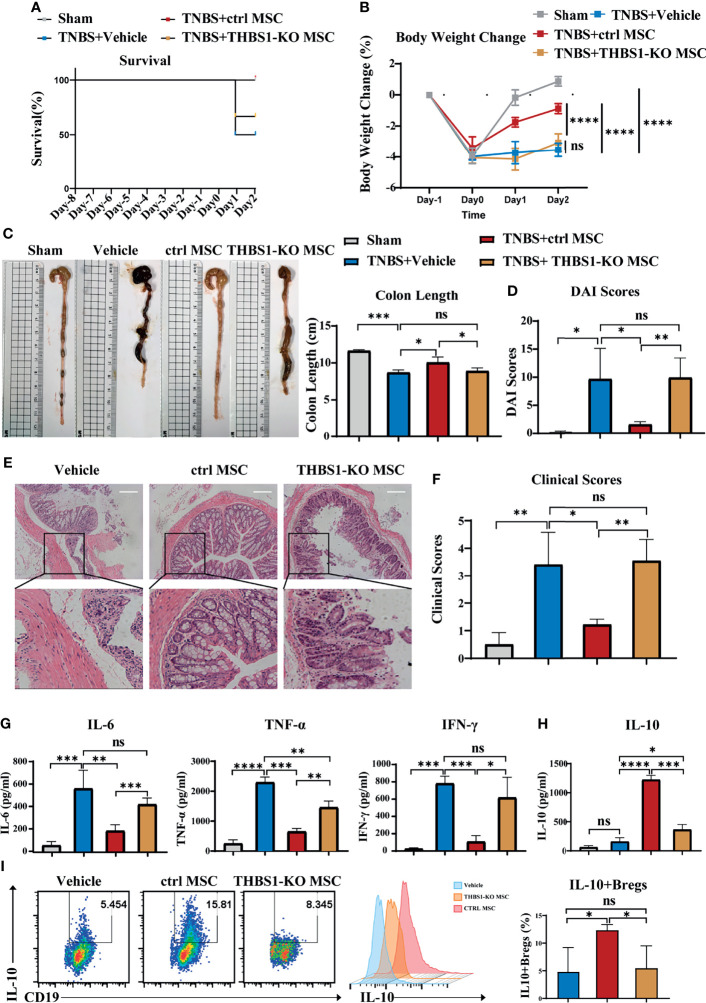
THBS1 plays a vital role in MSCs inducing IL-10^+^Bregs and protecting mice from colitis. THBS1-KO MSCs and control MSCs were separately injected into the peritoneal cavity of mice with colitis, and their therapeutic effects were evaluated. **(A)** Comparison of survival rate, **(B)** comparison of body weight loss, **(C)** representative colon graph and statistical colon length, **(D)** comparison of DAI scores. **(E)** Histological examination of colon cross sections *via* H&E staining and **(F)** histological scoring. **(G)** Colon tissue was collected, and the expression levels of proinflammatory cytokines in colon homogenate were detected by ELISA. **(H)** Peritoneal lavage fluid (including peritoneal cells) was collected, and the peritoneal fluid IL-10 level was determined by ELISA. **(I)** Peritoneal lavage fluid (including peritoneal cells) was collected, and the frequency of peritoneal IL-10^+^ Bregs was detected *via* flow cytometry. Data are presented as the means ± SD, n ≥ 3. not significant (ns) p≥0.05, *p < 0.05, **p < 0.01, ***p < 0.001, ****p < 0.0001.

To investigate whether THBS1 participates in MSC-mediated alleviation of colitis, the CRISPR-cas9 gene editing system was used to construct THBS1 knockout MSCs. First, the expression of THBS1 was further confirmed *via* flow cytometry detection, and the results showed no expression of THBS1 in THBS1-KO MSCs compared with control MSCs (**Figures S4A, B**). Knockout of THBS1 in MSCs did not change their characteristics (**Figure S1**). THBS1-KO MSCs and control MSCs were injected intraperitoneally to treat TNBS-induced colitis. Compared with the control MSC group, the survival rate (**Figure 5A**) was decreased and body weight loss (**Figure 5B**) of mice increased significantly when treated with THBS1-KO MSCs. Colon length (**Figure 5C**) and DAI score (**Figure 5D**) showed that THBS1-KO MSCs had little effect against the severity of colitis. HE results further confirmed that the MSC-mediated improvement in pathological colon inflammation in mice was almost reversed by deletion of THBS1 (**Figures 5E, F**). Accordingly, the levels of the proinflammatory factors IL-6, TNF-α, and IFN-γ were maintained at a high level in colon tissue when treated with THBS1-KO MSCs compared with control MSCs (**Figure 5G**). Since we demonstrated the critical role of IL-10-producing B cells in MSC-mediated alleviation of colitis, we further investigated whether THBS1 contributed to this effect. We found that control MSCs, but not THBS1-KO MSCs, significantly enhanced the level of IL-10 in the peritoneal cavity (**Figure 5H**). Peritoneal cell analysis showed that THBS1-KO MSCs lost the ability to induce the IL-10^+^Bregs in the peritoneal cavity (**Figure 5I**). For further verification, peritoneal cells were collected and cocultured with MSCs *in vitro*. As shown in **Figure S4C**, knockout of THBS1 in MSCs dramatically attenuated their effects on inducing IL-10^+^Bregs. Collectively, MSC-derived THBS1 could induce IL-10-producing B cells, which contributed to MSC-mediated therapeutic effects on colitis.

### MSCs Induced Peritoneal IL-10^+^ Bregs *via* THBS1-Dependent TGF-β Activation

**Figure 6:**
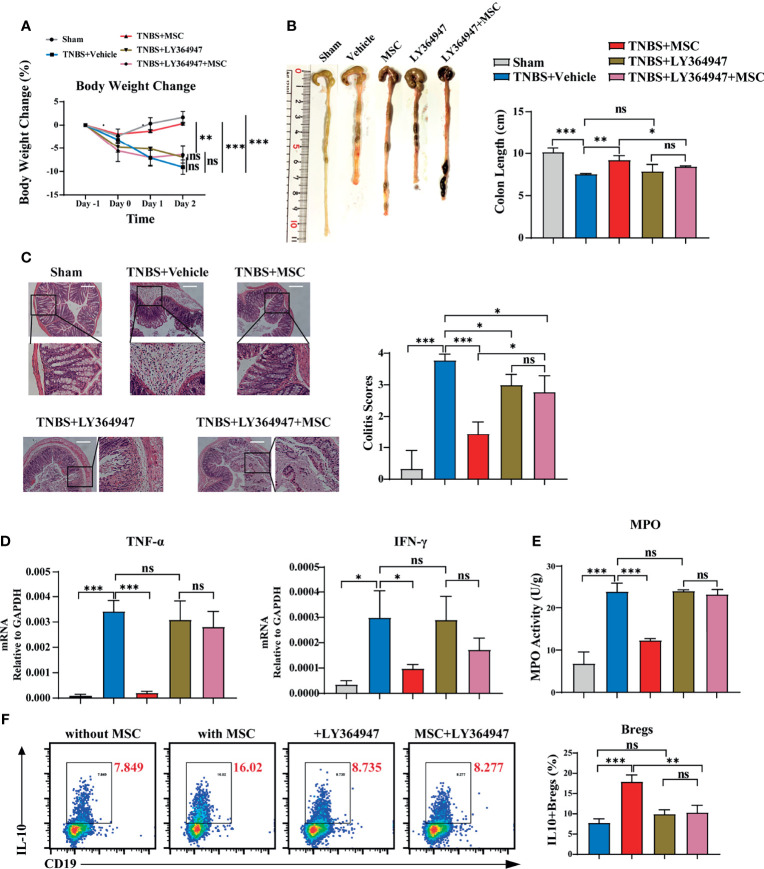
TGF-βR signalling participated critically in MSC-induced IL-10^+^Bregs to protect against TNBS-induced colitis. To investigate whether TGF-βR was involved, LY364947 (1 mg/kg) was intraperitoneally administered 3 times a week to block TGF-βR before TNBS enema and MSC administration. The alteration of MSC-mediated protection against colitis was evaluated. **(A)** Comparison of body weight loss. **(B)** Representative colon graph and statistical colon length. **(C)** Histological examination of colon cross sections *via* H&E staining and histological scoring. **(D)** Colon tissue was collected, and the expression levels of proinflammatory cytokines in colon homogenate were detected by ELISA. **(E)** Colon tissue was collected, and MPO activity was detected. **(F)** Peritoneal cells were collected and cultured with or without MSCs *in vitro* in the presence of LY364947 (10 μM) for 24 h IL-10^+^B cells were detected *via* flow cytometry. Data are presented as the means ± SD, n ≥ 3. not significant (ns) p≥0.05, *p < 0.05, **p < 0.01, ***p < 0.001, ****p < 0.0001.

THBS1 is a large extracellular matrix glycoprotein and can mediate cell–cell or cell–matrix interactions with a number of proteins, including α6β1 or α4β1 integrins and surface receptors such as CD36 and CD47 (43). However, when B cells were separated from MSCs using a transwell culture, the MSC-mediated effects on inducing IL-10-producing B cells were not obviously affected (**Figure S5**), indicating that direct interaction might not be the main mode of action by which THBS1 induces IL-10^+^B cells. A previous study reported that the type 1 domain of THBS1 is responsible for activating TGFβ1 by mobilizing its active from the potential activator protein (LAP) (44). Their interaction is between the KRFK sequence in thrombospondin and the LSKL sequence in LAP, which is important for the regulation of TGF-β activity both *in vitro* and *in vivo* (45). To investigate whether THBS1-mediated TGF-β activation was involved, LSKL peptide, a competitive antagonist for TGF-β1, was added to the coculture of peritoneal B cells and MSCs. Surprisingly, blocking TGF-β activation by LSKL almost reversed the MSC-mediated effects on inducing IL-10-producing B cells (**Figure S6**). Since TGF-β exerts its regulation of target cell function mainly *via* its receptor TGF-βR (46), we further evaluated the role of the TGF-β/TGF-βR system in MSCs treating colitis. LY364947, a common selective TGF-βR inhibitor for *in vitro* or *in vivo* studies (47), was injected into the peritoneal cavity before MSC administration in colitis mice. We found that LY364947 injection had little effect on the development of colitis. Compared to MSC injection alone, MSC injection combined with LY364947 did not effectively alleviate colitis, manifesting in weight loss (**Figure 6A**) and colon contractures (**Figure 6B**). HE results further confirmed that the MSC-mediated improvement in pathological colon inflammation in mice was almost reversed by blocking TGF-βR (**Figure 6C**). Accordingly, serious inflammatory cell infiltration (**Figure 6E**) and high levels of proinflammatory factors (**Figure 6D**) in colon tissue were observed in mice treated with MSCs while blocking TGF-βR, supporting that MSCs lost their function in controlling colitis in the absence of the TGF-β/TGF-βR system. More importantly, in the presence of LY364947, IL-10^+^Bregs could not be induced by MSCs (**Figure 6F**), proving that MSCs induced peritoneal IL-10^+^Bregs *via* THBS1-dependent TGF-β activation.

## Discussion

MSCs have become a promising treatment for IBD, even for those who are refractory to conventional therapies (48). Although many clinical studies have demonstrated the safety and efficacy of MSCs in controlling IBD, there are still some conflicting results reported and even clinical trial failure (49). MSC therapeutic potency depends upon viability, route of delivery, immune match, etc. (50). An increasing number of studies have revealed that the delivery route is a key influencing factor for MSC-mediated effects in treating IBD. Intravenous injection of MSCs might not be an effective delivery method for IBD and easily yields unsatisfactory outcomes (51). Local intralesional injection of MSCs showed favourable effects for healing perianal fistulas in patients with IBD but might be inconvenient for patients without perianal fistulas (52). In experimental colitis, Giri reported that intraperitoneal delivery of MSCs is more effective than intravenous injection in reducing colitis severity (50). Similarly, we found that intraperitoneal injection of MSCs inhibited the severity of colitis in mice more effectively. More importantly, intraperitoneally delivered MSCs also exerted their predominant effects in preventing the recurrence of colitis. These are very important for IBD management, as routine treatments are still difficult to control or inhibit the recurrence of IBD (53). Considering that intraperitoneal injection has shown benefits in many human and animal studies, even as recommended by the National Cancer Institute (NCI) for advanced ovarian chemotherapy (24, 54), it is worth trying MSC delivery *via* intraperitoneal injection for IBD treatment in the clinic.

Local injection of MSCs exerts better effects, which is mainly due to the large number of MSCs at the injury site (55). However, intraperitoneally delivered MSCs rarely migrate to the intestinal tissue (25). Their therapeutic effects are likely due to the unique microenvironment modulated by MSCs in the peritoneal cavity. The peritoneal microenvironment plays an important role in the course of colitis (27). Peritoneal cavity resident B cells and macrophages are both associated with IBD (56, 57). Jinju Kim and colleagues proved that depletion of macrophages by clodronate liposomes attenuated colon inflammation (58). Similar to our results, we found that peritoneal macrophages participated in the development of colitis; however, the depletion of macrophages had little effect on the MSC-mediated therapeutic effects on colitis. Compared to macrophage deletion, the protective function of MSCs against colitis was severely impaired after B cell depletion, suggesting the important role of B cells in the alleviation of colitis in mice by MSCs. B cells are critical for immune homeostasis, including the gastrointestinal tract (59). B cell-related abnormalities such as lymphoplasmacytic inflammatory infiltration are well-established pathological hallmarks of IBD (60). However, attempts to target B cell immunity, especially rituximab, an anti-CD20 monoclonal antibody that depletes pan-B cells, have proven unsuccessful in clinical trials (61). A limitation of mucosal B cell depletion by anti-CD20 has also been mentioned: indiscriminate targeting of all B cells, such as regulatory B cells, has no benefit in remission of the disease (62). In a previous study, B cells from patients with Crohn’s disease (CD) had significantly reduced IL-10 production induced by CpG DNA and were effective in the treatment of Crohn’s disease (CD) by adoptive transfer experiments after *in vitro* induction of IL-10^+^ Bregs (63). We further demonstrated that MSCs likely boosted IL-10-producing B cells, which mediated the improvement in colitis. Regulatory B cell subsets expressing IL-10 (B10 cells) modulate immune responses and the severity of autoimmune diseases (64, 65). The peritoneal cavity contains a relatively high frequency of functionally defined IL-10^+^ B cells, and the IL-10 produced by peritoneal B cells significantly reduces disease severity in both spontaneous and induced colitis models by modulating neutrophil infiltration during colitis episodes, colonic-induced CD4^+^ T cell activation, and proinflammatory cytokine production (57). More importantly, we found that the increase in IL-10-producing B cells in the peritoneal cavity was also beneficial for preventing the recurrence of colitis. These findings might provide new strategies of cell therapy for IBD treatment or prevention.

THBS1 is a member of the matricellular protein family that is important in the control of extracellular matrix (ECM) remodelling and has also been reported to promote the proliferation and migration of mesenchymal cells through modulating TGFβ activation or protecting the degradation of PDGF (66, 67). There are many studies on the MSC-secreted THBS1 in regulating angiogenesis, but rarely on its immunoregulatory function in MSCs (68, 69). Marie Maumus and colleagues reported that THBS1 secreted by adipose-derived MSCs may play a protective role in a collagen-induced osteoarthritis (CIOA) mouse model by chondroprotective effects and anti-inflammatory effects on T lymphocytes (70). Here, we found that THBS1 plays an important role in MSC protection against colitis in mice, as the absence of THBS1 in MSCs significantly reversed their immune regulation. Furthermore, we proved for the first time that THBS1 is involved in the induction of IL-10^+^Bregs and proposed that MSCs could exert immunoregulatory functions through THBS1. THBS1 was also reported to trigger IL-10 production in macrophages (42) and inhibit TCR-mediated T cell activation (71), suggesting that THBS1 might be an important immune modulator. THBS1 modulates immune cells likely *via* its receptors, such as CD36 and CD47 (42, 71). Here, we revealed that MSC-derived THBS1 induced IL-10-producing B cells mainly *via* its effects on controlling TGF-β activation, an indirect way for immunomodulation. THBS1-deficient mice exhibited inflammation, with a phenotype similar to that observed in TGFβ-deficient mice, and a more severe course of acute colitis (72), further supporting the critical role of THBS1 and activating TGF-β in modulating colitis. Furthermore, we demonstrated the loss of protective function of MSCs against acute colitis in mice by blocking TGF-βR, accompanied by the induced loss of IL-10^+^Bregs. Similar results were reported that when blocking TGF-β or IL-10, peritoneal cell-mediated colitis improvements were lost (27), confirming the important role of TGF-β or IL-10 in peritoneal cells to exert their therapeutic effects. TGF-β is abundant in the mammalian intestine and is produced by many cell types, including epithelial cells, immune cells, and fibroblasts (73); however, the source of TGF-β in the peritoneal environment and its alteration during colitis should be further elucidated. Nevertheless, our results provide new clues to unravel the MSC-mediated immunoregulatory mechanism for IBD and new targets for IBD treatment and prevention.

## Conclusion

In this study, we explored the underlying mechanism by which intraperitoneally delivered MSCs alleviated colitis and found that MSC-derived THBS1 could boost IL-10-producing B cells through THBS1-mediated TGF-β activation, thereby suppressing colon inflammation and preventing the recurrence of colitis. These results provide new insight for the prevention and treatment of IBD.

## Data Availability Statement

The original contributions presented in the study are publicly available. These data can be found here: https://www.ncbi.nlm.nih.gov/bioproject/PRJNA801422.

## Ethics Statement

The animal study was reviewed and approved by Sun Yat-sen University Institutional Animal Care and Use Committee.

## Author Contributions

JL, XL, and YB designed the experiments, performed the research, interpreted the data, and wrote the manuscript. WX performed for data mining and wrote the manuscript. ZL constructed the plasmid and collected the animal samples. JC, GL, and TW participated in performing the animal models. WH and YM participated in the data analysis. JS and EZ participated in the research. APX, QL, and XC conceptualized and designed the study, supervised the research, interpreted the data, and wrote the manuscript. All authors contributed to the article and approved the submitted version.

## Funding

This work was supported by grants from the National Key Research and Development Program of China, Stem Cell and Translational Research (2017YFA0105501, 2018YFA0107203); the National Natural Science Foundation of China (81730005, 81971526, 81970109); the Key Scientific and Technological Projects of Guangdong Province (2019B020236004, 2019B020234001, 2019B020235002, 2017B020230004, 2015B020226002); Guangdong Basic and Applied Basic Research Foundation (2020A1515010272); and Key Scientific and Technological Program of Guangzhou City (201803040011).

## Conflict of Interest

The authors declare that the research was conducted in the absence of any commercial or financial relationships that could be construed as a potential conflict of interest.

## Publisher’s Note

All claims expressed in this article are solely those of the authors and do not necessarily represent those of their affiliated organizations, or those of the publisher, the editors and the reviewers. Any product that may be evaluated in this article, or claim that may be made by its manufacturer, is not guaranteed or endorsed by the publisher.
