# Supplementary material for: Intraperitoneally Delivered Mesenchymal Stem Cells Alleviate Experimental Colitis Through THBS1-Mediated Induction of IL-10-Competent Regulatory B Cells
Source: Front Immunol. 2022 Mar 18;13:853894. doi: 10.3389/fimmu.2022.853894 (PMC8971528; doi:10.3389/fimmu.2022.853894)
Supplement: Supplementary file 1 [file DataSheet_1.pdf]

Supplementary Material

Supplementary Figure 1.

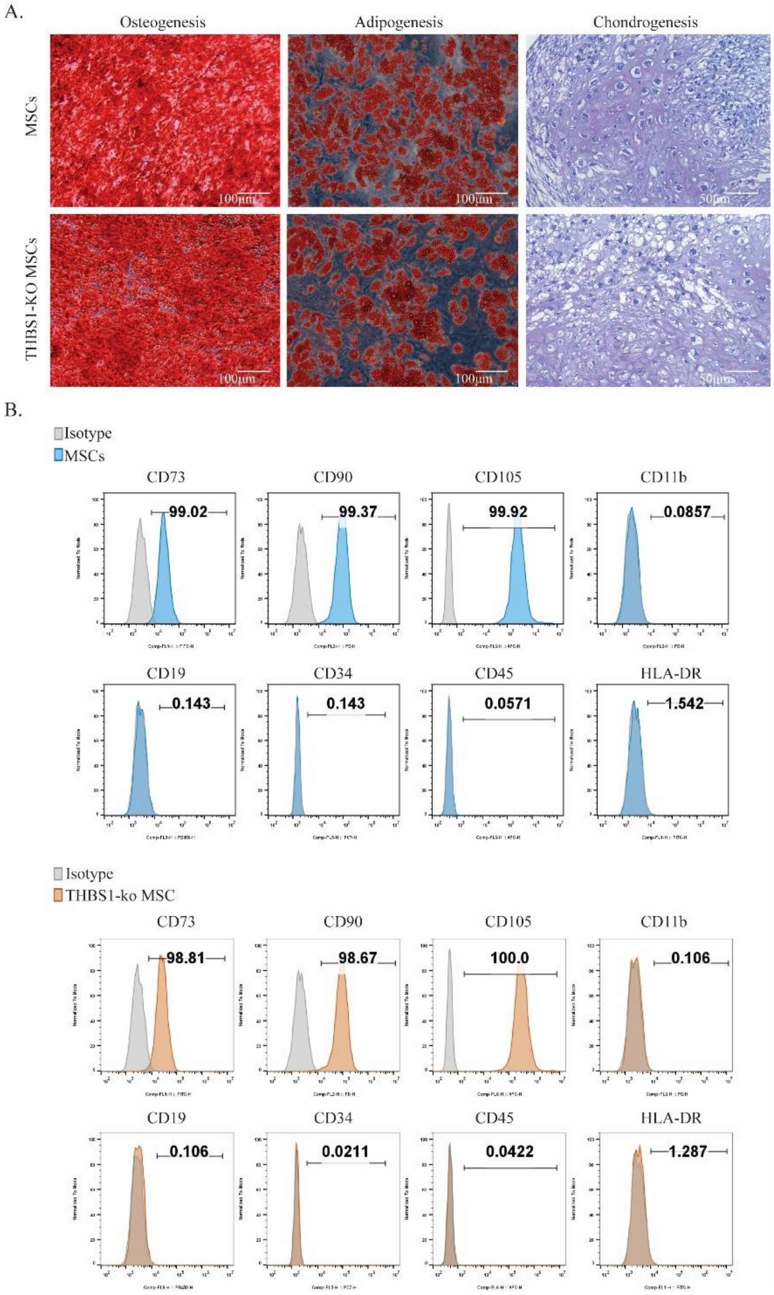

Figure S1. Identification of Human Bone morrow MSCs. (A) Oil red O, Alizarin red S, and Toluidine blue O staining were used to assess the adipogenic, osteogenic, and chondrogenic differentiations of MSCs. (B) The expression of CD73, CD90, CD105, CD11b, CD19, CD34, CD45 and HLA-DR on MSCs were detected by Flow Cytometry.

Supplementary Figure 2.

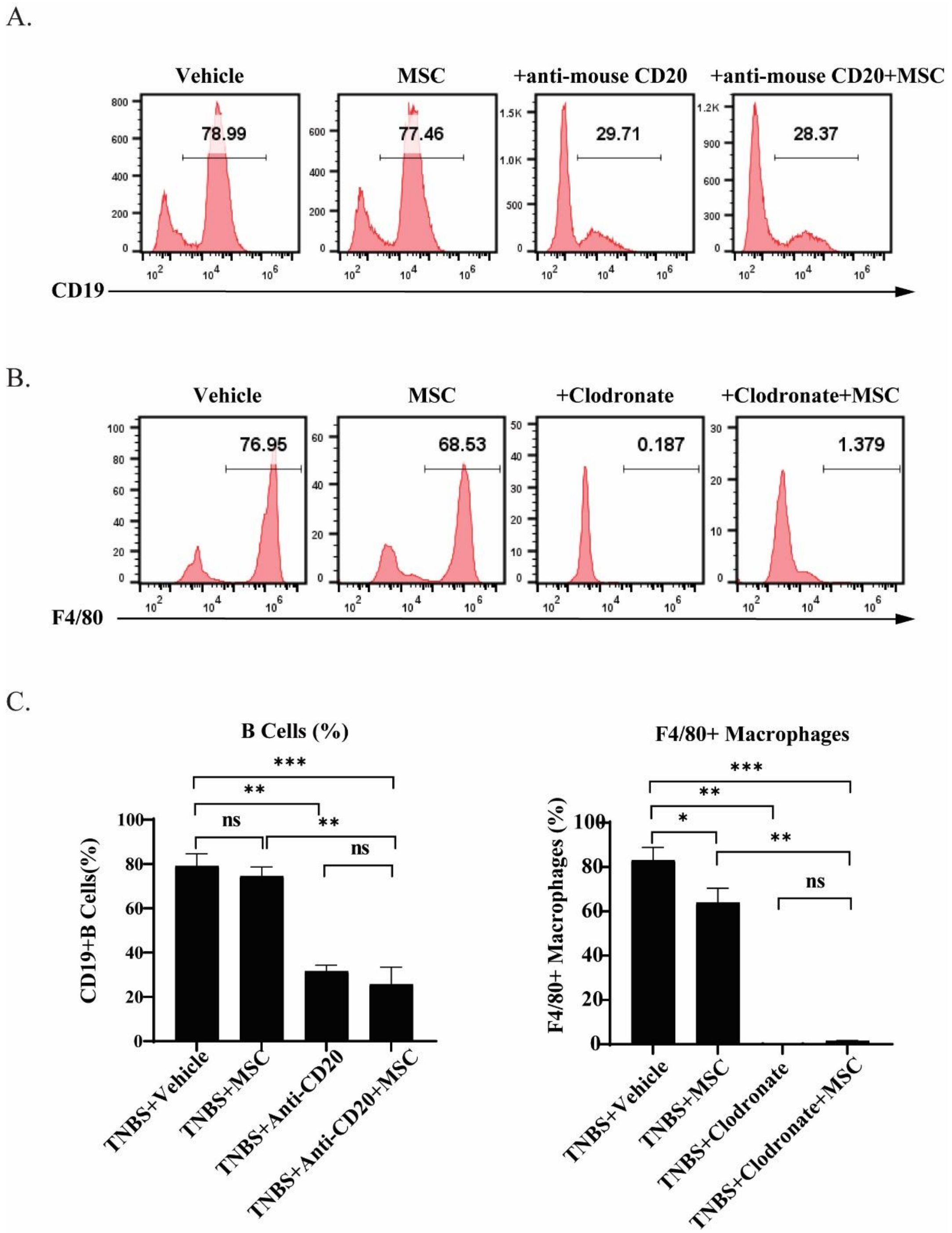

Figure S2. The Alteration of B cells and macrophage in Peritoneal Cavity. (A) Representative plots of alteration in percentages of B cells in peritoneal cavity. (B) Representative plots of alteration in percentages of macrophage in peritoneal cavity. (C) Quantification of B cells and macrophage in peritoneal cavity. Data are mean ± SD; not significant (ns) p⩾0.05, *p<0.05, **p<0.01, ***p<0.001, ****p<0.0001.

Supplementary Figure 3.

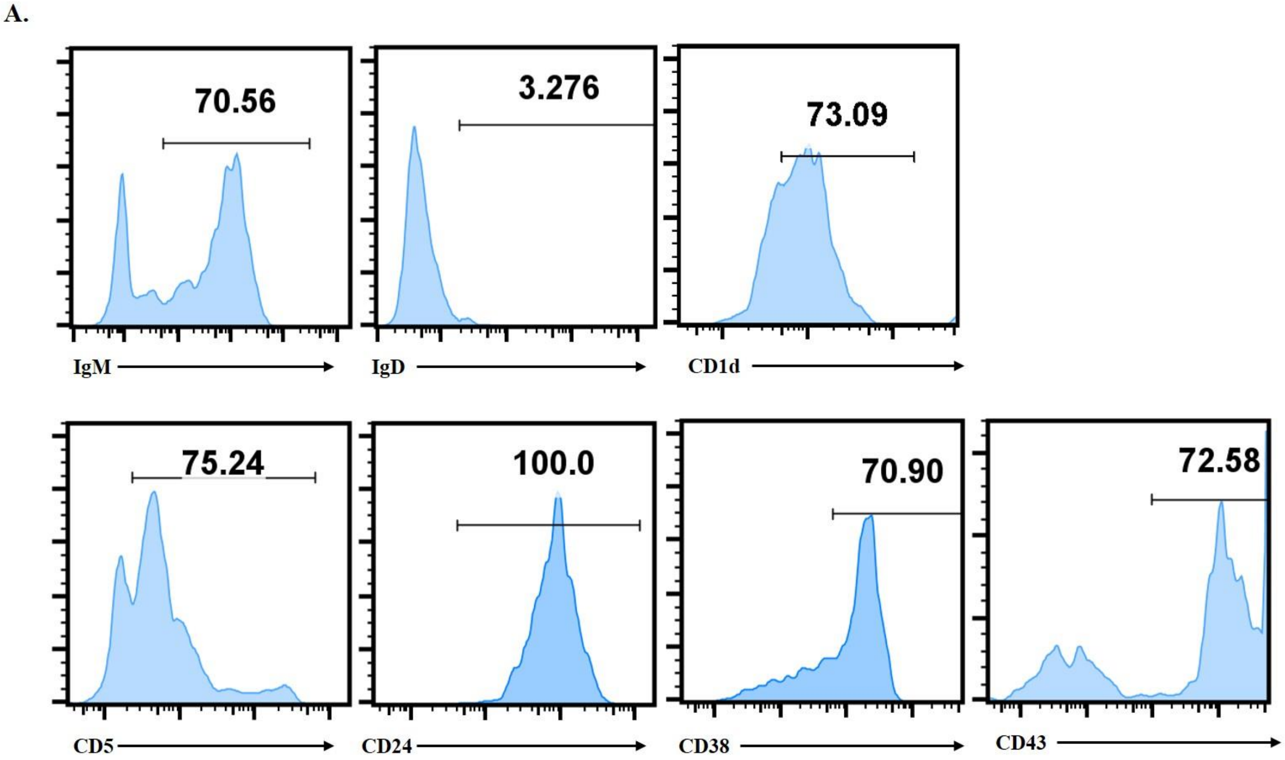

Figure S3. Surface Phenotype of MSC-induced IL-10$^+$Bregs. Peritoneal B cells were isolated from colitic mice with MSCs treatment, and the phenotype of IL-10$^+$Bregs were detected, including IgM, IgD, CD1d, CD5, CD24, CD38, CD43.

Supplementary Figure 4.

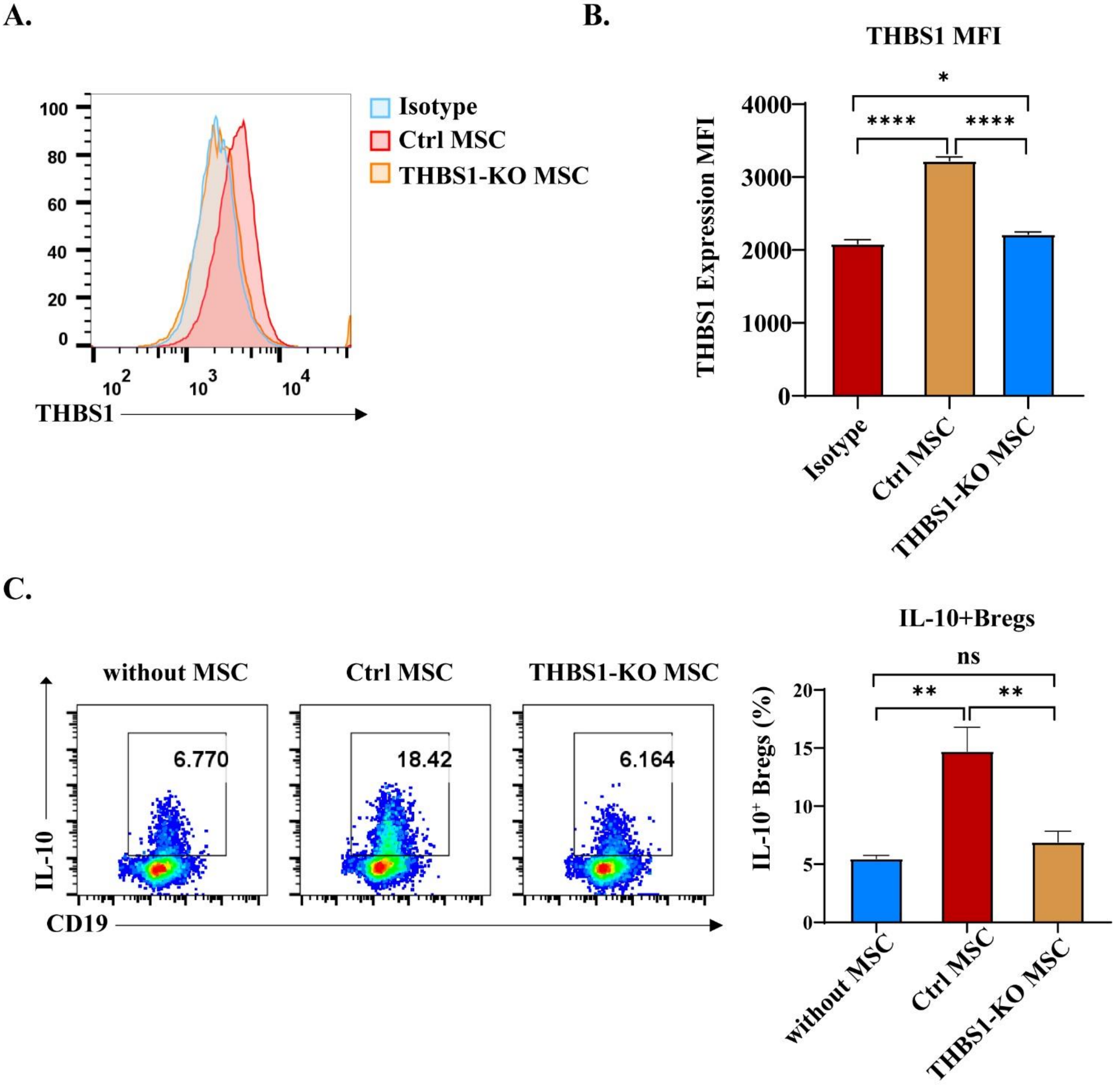

Figure S4. THBS1 Knockout in MSCs impaired their capacity to induce IL-10 producing Bregs in vitro. (A) The expressions of THBS1 were detected by flow cytometry. (B) Quantification of the Mean Fluorescence Intensity (MFI) of THBS1 both in control MSCs and THBS1-KO MSCs. (C) The alterations of IL-10 producing B cells. Data are mean ± SD, n⩾3, not significant (ns) p⩾0.05, *p<0.05, **p<0.01, ***p<0.001, ****p<0.0001.

Supplementary Figure 5.

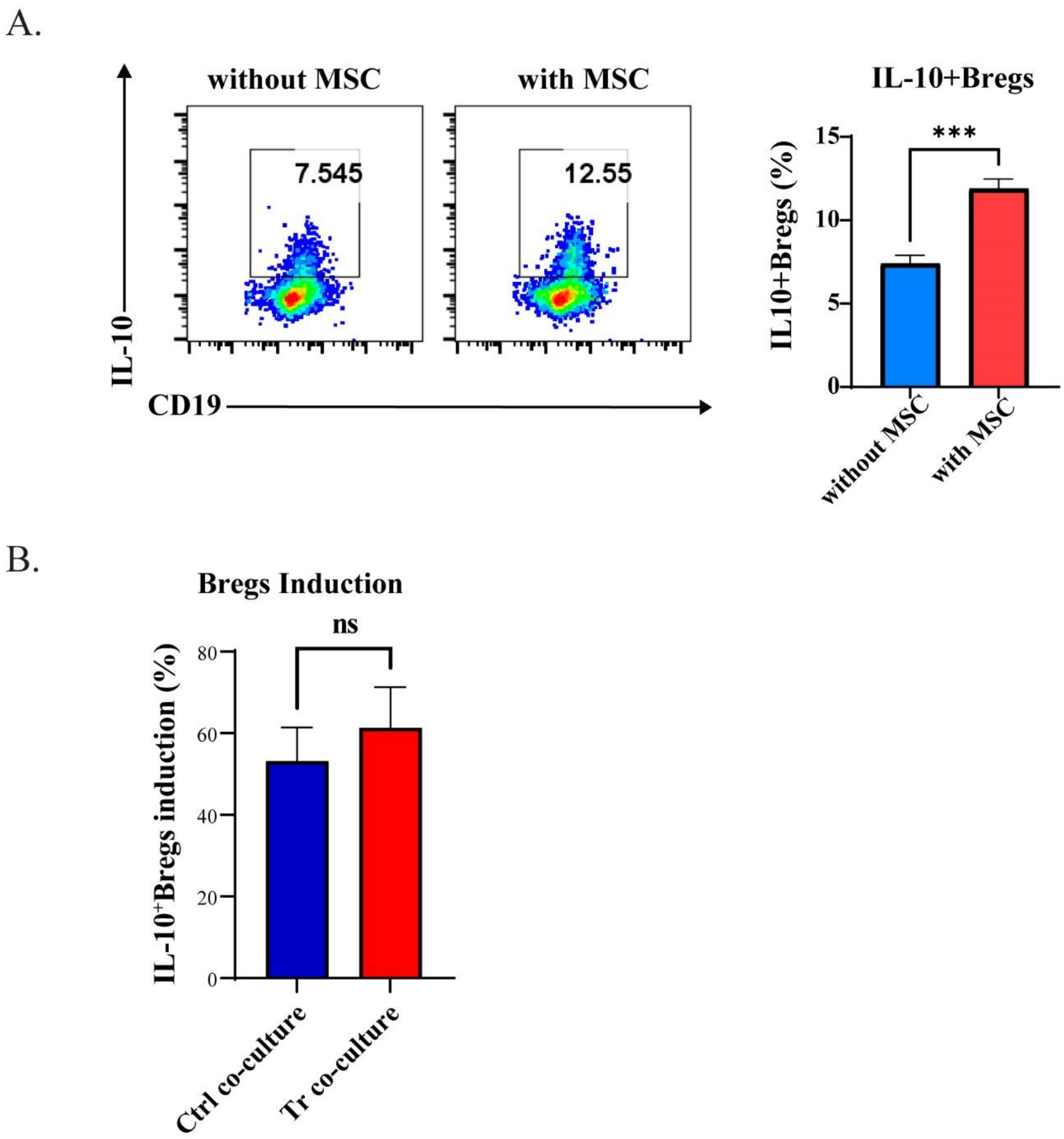

Figure S5. MSCs induced IL-10⁺ Bregs in Transwell indirect co-culture. (A) IL-10⁺Bregs detection by Transwell indirect co-culture 48hours between MSCs and mice peritoneal cells. (B) IL-10⁺Bregs induction of MSCs in control direct co-culture (Ctrl co-culture) compared with Transwell indirect co-culture (Tr co-culture). Data are mean ± SD, n⩾3, not significant (ns) p⩾0.05, *p<0.05, **p<0.01, ***p<0.001, ****p<0.0001.

Supplementary Figure 6.

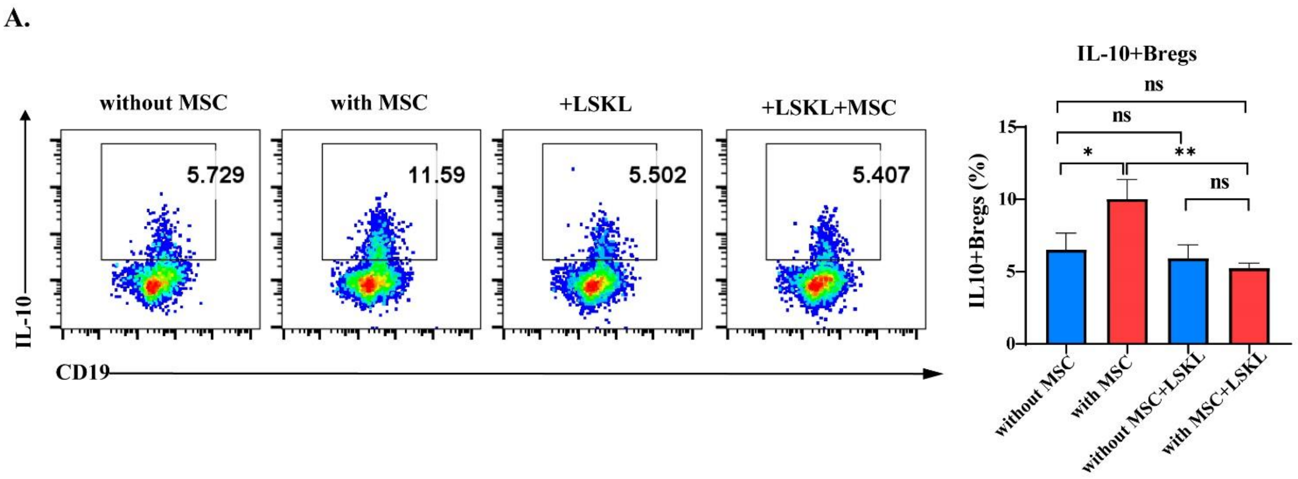

Figure S6. LSKL Treating MSCs induced IL-10+ Bregs in vitro. (A) IL-10+Bregs detection by MSCs co-culture and LSKL treated after 48 hours. Statistical significance determined by unpaired two-sided Student's t test. Data are mean ± SD, n⩾3, not significant (ns) p⩾0.05, *p<0.05, **p<0.01, ***p<0.001, ****p<0.0001.

Supplement material Table 1.

Antibody resources table

Antibody	Catalog	Manufactory
FITC Mouse Anti-Human CD73	561254	BD Biosciences
PE Mouse anti-Human CD105	560839	BD Biosciences
APC Mouse Anti-Human CD90	561971	BD Biosciences
BV421 Mouse Anti-Human CD19	562440	BD Biosciences
PE Mouse Anti-Human CD11b	555388	BD Biosciences
PE-Cy™7 Mouse Anti-Human CD34	560710	BD Biosciences
APC Mouse Anti-Human CD45RA	561210	BD Biosciences
FITC Mouse Anti-Human HLA-DR	555811	BD Biosciences
PE-Cy™7 Rat Anti-Mouse CD19	552854	BD Biosciences
FITC Rat Anti-Mouse CD5	553021	BD Biosciences
BV421 Rat Anti-Mouse F4/80	565411	BD Biosciences
APC Rat Anti-Mouse IL-10	554468	BD Biosciences
APC Rat Anti-Mouse IgM	550676	BD Biosciences
V450 Rat Anti-Mouse IgD	560869	BD Biosciences
PE Rat Anti-Mouse CD1d	12-0011-82	Invitrogen
FITC Rat Anti-Mouse CD24	11-0242-82	Invitrogen
PE Rat Anti-mouse CD38	102707	BioLegend
PE Rat Anti-mouse CD43	553271	BD Biosciences
Anti-Thrombospondin 1 antibody	ab1823	Abcam
Goat anti-Mouse IgG (H+L) Cross-Adsorbed ReadyProbes™ Secondary Antibody, Alexa Fluor 594	R37121	Invitrogen
